# Rapid and Continuous Preparation of Polyacrylonitrile-Based Carbon Fibers with Electron-Beam Irradiation Pretreatment

**DOI:** 10.3390/ma11081270

**Published:** 2018-07-24

**Authors:** Jia Yang, Yuchen Liu, Jie Liu, Zhigang Shen, Jieying Liang, Xiaoxu Wang

**Affiliations:** 1 Key Laboratory of Carbon Fiber and Functional Polymers, Ministry of Education, Beijing University of Chemical Technology, Chao-Yang District, Beijing 100029, China; yangjcarbon@163.com (J.Y.); 13701098376@163.com (Y.L.); liangjy@mail.buct.edu.cn (J.Liang); 2 SINOPEC Shanghai Research Institute of Petrochemical Technology, 1658 Pudong North Road, Pudong District, Shanghai 201208, China; shenzg.sshy@sinopec.com

**Keywords:** carbon fibers, rapid stabilization, electron-beam irradiation, continuous production

## Abstract

Thermal stabilization is a critical, yet time- and energy-consuming process during the preparation of PAN-based carbon fibers. In this work, automobile-grade carbon fibers with a 2.85 GPa tensile strength and a 203 GPa modulus are continuously produced with electron-beam (e-beam) irradiation pretreatment and 24 min thermal stabilization. Thermal and structural analyses reveal that e-beam irradiation can lower the onset temperature of the cyclization reaction and mitigate the heat release. Meanwhile, during the process of stabilization, e-beam irradiation can facilitate the evolution of both the chemical structure and the crystalline structure of polyacrylonitrile (PAN) fibers. Comparing to the industrial production of carbon fiber with a 40 min stabilization time, e-beam irradiated PAN fibers can achieve the same degree of stabilization with a 40% time savings.

## 1. Introduction

Carbon fiber composites have gained a wide range of applications from sporting products to the aerospace industry [1,2]. In recent years, there is an increasing demand for low-cost carbon fibers in the automobile industry, since the majority of automotive manufacturers have carried out extensive vehicle weight reduction plans, owing to stringent environmental regulations [3]. Carbon fiber can offer a much higher strength to weight ratio compared to steal and aluminum and has found success in the weight reduction of vehicles. However, the automobile industry does not require the ultra-high mechanical properties of carbon fibers, but is rather sensitive to the cost [4]. Accordingly, the U.S. Department of Energy (DOE) has made the development of low-cost automotive-grade carbon fiber its highest priority for materials research [5]. The DOE’s targeted mechanical properties of automobile-grade carbon fibers are a 1.72 GPa tensile strength and a 172 GPa modulus. Meanwhile, the DOE’s targeted price of automobile-grade carbon fiber is $11–$15.4/kg, which is around one order of magnitude lower than current commercial carbon fibers [6]. Considering the tremendous potential market in the automobile industry, a significant amount of industrial and academic efforts have been devoted to lower the cost of carbon fibers. 

Polyacrylonitrile (PAN) has been viewed as the most suitable precursor for making carbon fibers among pitch, rayon and cellulose [7]. Commercial PAN-based precursor materials are commonly produced by using polymerization mixtures of AN with more than one co-monomer, for example with methyl acrylate and itaconic acid, to increase the spinnability and mitigate the heat evolution [1,8]. The manufacture of PAN-based carbon fibers typically involves three steps: spinning of PAN precursor fibers, thermal stabilization and carbonization. The thermal stabilization process is the most critical step during which the linear PAN chains convert to thermally-stable ladder-like structures; this step ensures efficient conversion of polymer to carbon with high structural integrity and carbon yield [9]. This process consists of a series of sophisticated chemical reactions such as cyclization of the nitrile groups, dehydrogenation of the cross-linking chain molecules, as well as oxidation reaction [7]. The thermal stabilization reaction is exothermic, and the sudden evolution of heat can partially melt or even ignite the fiber [10]. Thus, the reaction rate of stabilization has to be restricted by lowering the reaction temperature. The stabilization of PAN is also a diffusion-controlled process, which is a function of stabilization time [11]. The large diameter of PAN fibers delays the diffusion of molecules including oxygen and stabilization by-products between the skin and the core of the fiber, and the oxidation on the outer region of the fiber would generate a dense layer and hinder further diffusion of oxygen into the inner region [12]. As the result of the slow reaction rate and hindered diffusion process, thermal stabilization takes a long time (~1 h) and becomes one of the most time- and energy-consuming steps during the preparation of carbon fibers. Thus, among various methods for lowing the production cost of carbon fibers, new oxidation methods using radiation, plasma or ultra-violet treatments to shorten stabilization time have become of great interest in recent years [13].

Electron-beam (denoted as e-beam) irradiation is a cost-effective and environmentally-friendly method that has been widely used in industry for cable insulation crosslinking and sterilizing applications [14]. As early as 1995, Hirt and co-workers had successfully applied e-beam irradiation on PAN fibers to reduce the stabilization time, and they revealed that the e-beam can initiate radical-induced cyclization of PAN polymer chains [9]. Since then, many efforts have been devoted to study the effect of e-beam irradiation on the stabilization behavior of PAN fibers [2,15,16,17,18,19]. However, successful preparation of carbon fibers based on e-beam irradiation pretreatment is rarely reported, and the application of e-beam irradiation to industrial production of carbon fibers is delayed. Shin et al. prepared carbon fibers based on the combination of e-beam irradiation and short thermal treatment, wherein PAN fibers, irradiated by 1000 kGy, were thermally oxidized at 200 °C or 250 °C for 20 or 40 min, respectively [16]. The results of Fourier transform infrared spectrometer (FT-IR) analysis showed that the C≡N peak at 2244 cm^−1^ almost disappeared after 40 min at 250 °C, while the intensity of the C=N peak at 1628 cm^−1^ was indicative of an increase in cyclization. Through differential scanning calorimeter (DSC) analysis, thermal treatment at either 200 °C or 250 °C was found to cause a sharp decrease in the majority of exothermic peaks. The tensile strength of resulting carbon fibers was around 2.3 GPa. Recently, Park and co-workers carried out a comprehensive stabilization mechanism study of e-beam-irradiated PAN fibers, and they successfully prepared carbon fibers with a tensile strength and modulus of 2.3 and 216 GPa, respectively, based on a 30 min stabilization time [20]. However, these carbon fibers were prepared based on a batch process, which was typically finished by placing PAN fibers in a cart under the e-beam irradiation source. In practice, research outcomes based on the continuous production method would be more applicable for industrial production of carbon fibers.

In this study, a 12k carbon fiber tow is continuously prepared with the process of e-beam irradiation pretreatment, thermal stabilization and carbonization using a pilot-scale (five tons/year) production line. The effects of e-beam irradiation on the thermal and structural evolution of PAN fibers during stabilization are studied using DSC, FTIR, X-ray diffraction (XRD) and bulk density measurements. Compared to the current industrial carbon fiber production with a 40-min stabilization time, e-beam-irradiated PAN fibers are stabilized in under 24 min, and their degree of stabilization is compared based on density and FT-IR measurements. Finally, the mechanical properties of carbon fibers with irradiation pretreatment are characterized and compared with non-irradiated carbon fibers.

## 2. Materials and Methods 

### 2.1. Materials

PAN fibers used in this study were SAF 12K PAN copolymer fibers with 92.8 wt% of acrylonitrile (AN), 1.2 wt% of itaconic acid (IA) and 6.0 wt% of methyl acrylate (MA) provided by the Courtaulds, Ltd. UK. Each tow of the fibers consisted of 12,000 filaments.

### 2.2. Electron Beam Irradiation of PAN Fibers

PAN precursor fibers were continuously irradiated at room temperature with an electron accelerator in air provided by CYG Electronics Co., Ltd. The equipment setup can be found in the Supporting Information, Figure S1. The acceleration voltage used in the experiment was 1.5 MeV, and the electron beam current was 35 mA. PAN fibers were continuously passed under the acceleration gun. The irradiation dose of 800 kGy was determined by the irradiation time of 20 s, which was controlled by the fiber travel speed of 6 m/min.

### 2.3. Stabilization and Carbonization

**Figure 1:**
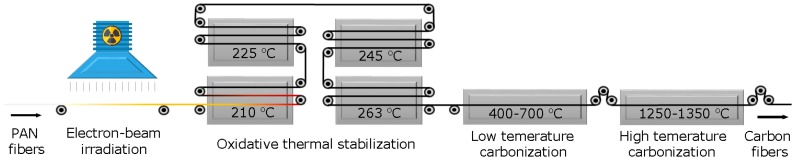
Schematic representation of the production line.

Carbon fibers from PAN and irradiated PAN precursors were prepared using a self-designed pilot production line, which consisted of four oxidizing ovens, one low-temperature carbonization furnace and one high-temperature carbonization furnace. The schematic representation of the production line is shown in Figure 1. The temperature of four stabilization ovens was programed as 210–225–245–263 °C with draw ratios of 2–0–0–0, respectively. The stabilization time was controlled by the travel distance of precursor fibers within the ovens. Carbonization was performed in a N_2_ atmosphere. The low-temperature carbonization furnace was divided into five temperature zones, with a temperature setup of 400–700 °C. The high-temperature carbonization furnace was also divided into five zones with a temperature setup of 1250–1350 °C.

### 2.4. Characterizations

Differential scanning calorimetry (DSC) conducted in air atmosphere was used to monitor the thermal behavior of two kinds of fibers by a METTLER DSC-1 differential scanning calorimeter. The range of heating temperature was 180–400°C, and different heating rates of 2 °C/min, 5 °C/min, 8 °C/min and 10 °C/min were applied for each sample. The activation energy (*E_k_*) of precursor fibers was calculated from the following Kissinger equation:

$$\ln\frac{\beta }{T^{2}}=\ln\frac{AR}{E_{k}}-\ln\frac{E_{k}}{R}-\frac{1}{T_{pk}}$$

where *E_k_* is the activation energy (J/mol), *β* is the heating rate (K/min), *T_pk_* is the temperature of the exothermic peak (K) and *R* is the ideal gas constant (8.314 J/mol·K).

A Lloyd DC-2 density gradient tube was adopted to measure the bulk density of the stabilized fibers and the carbon fibers. 

The X-ray diffraction (XRD) was conducted to characterize the crystalline structure evolution of both precursor fibers using a D/Max-2500 PC X-ray diffractometer with Cu Kα (*λ* = 0.1542 nm) radiation at a voltage of 40 Kv, a current of 45 mA and a scanning range from 5°–60°.

Fourier transform infrared spectroscopy (FT-IR) with a Nicolet 8700 spectrometer was used to analyze the functional groups of irradiated and untreated PAN fibers. Scanning from 500–4000 cm^−1^ with a resolution of 4 cm^−1^ was employed to examine the chemical changes during stabilization. The degree of relative cyclization (*RCI*(%)) was calculated as $RCI\left(\%\right)=\frac{I_{C\equiv N}}{I_{C\equiv N}+I_{C=N}}\times 100\%$, where $I_{C\equiv N}$ is the peak intensity of the C≡N vibration at 2243 cm^−1^ and $I_{C=N}$ is the peak intensity of the C=N vibration at 1591 cm^−1^.

The mechanical properties of carbon fibers were tested on the basis of the D4018–99 standardusing an INSTRON-5567 universal testing machine at a crosshead speed of 10.0 mm/min with a gage length of 150 mm. Eight specimens were tested for each type of carbon fiber. During the specimen preparation, each type of carbon fibers was wound onto a rectangular framework in parallel, and the aligned fibers were impregnated into a mixed solution (the mass ratio of acetone, epoxy 618 resin and triethylenetetramine was set at 20:10:1). Then, samples were cured in an oven at 120 °C for 2 h.

## 3. Results

**Figure 2:**
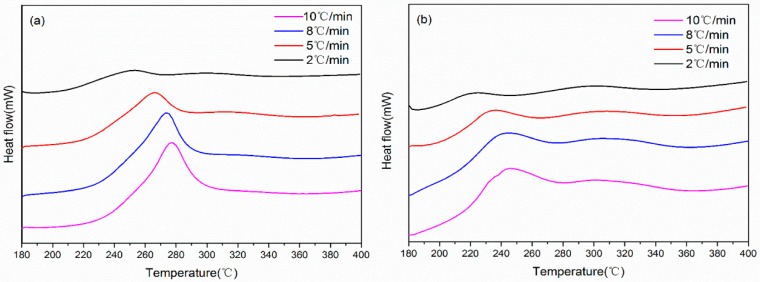
DSC curves of (**a**) PAN and (**b**) irradiated PAN fibers at different heating rates of 2, 5, 8 and 10 °C/min in air atmosphere.

The objective of using e-beam irradiation on PAN fibers is to reduce their thermal stabilization time. In order to identify the appropriate stabilization parameters for irradiated PAN fibers, differential scanning calorimetry (DSC) was first employed to study the thermal behavior of irradiated and non-irradiated PAN fibers. Figure 2 displays the exothermic curves of irradiated and non-irradiated PAN fibers at different heating rates of 2, 5, 8 and 10 °C/min in air atmosphere. The comparison of DSC curves for PAN and irradiated PAN fibers can be found in the Supporting Information, Figure S2. In general, the first exothermic peak corresponded to the cyclization reaction [21]. The DSC curves of untreated PAN fibers exhibited a narrow and sharp exothermic peak, indicating concentrated heat release. On the other hand, the DSC curves of irradiated PAN fibers showed a flat and broadened exothermic peak; this indicated that e-beam irradiation pretreatment can mitigate the exothermic cyclization reaction of PAN fibers. To quantitatively investigate the thermal behavior of irradiated PAN fibers, the onset temperature of exothermic reaction (*T_onset_*), the first exothermic peak temperature (*T_p._*_1_) and the enthalpy change (Δ*H*) were extracted from DSC curves and summarized in Table 1. The *T_onset_*, *T_p,1_* and the baseline for calculating Δ*H* are shown in the Supporting Information, Figure S3. Noticeably, the first exothermic peak (*T_p._*_1_) of PAN fibers shifted more than 30 °C to a lower temperature after irradiation, and the irradiated PAN fibers released much less heat during the measurement. Similar observations have been reported by several researchers, and they reveal that the e-beam irradiation can weaken the dipole force of nitrile groups and produce free radicals to enhance the abilities of cyclization between PAN molecules [16,22].

The activation energy (*E_k_*) of the cyclization reaction was calculated using the Kissinger method [23]. The *E_k_* values served to highlight the differences in DSC profiles observed between these two samples, and the *E_k_* of PAN fiber decreased from 119 kJ/mol–105 kJ/mol after irradiation. The lower *E_k_* of irradiated PAN fiber indicated that irradiation can facilitate the cyclization reaction of nitrile groups; therefore, the thermal stabilization can proceed with a shorter period of time. Thus, we can design stabilization parameters with less stabilization time. 

During thermal stabilization, both untreated PAN and irradiated PAN fibers were stabilized continuously through four temperature zones, and the degree of stabilization was controlled through adjusting the temperature and the dwelling time in each zone. To have a better understanding of the effect of dwelling time, the temperature of each zone was kept identical for both samples. Thus, the only difference of stabilization conditions between PAN and irradiated PAN fibers was the dwelling time in each zone. For untreated PAN fibers, the total stabilization time was 40 min with 10 min in each zone. The stabilization of 24 min (6 min in each zone) for irradiated PAN fiber was applied based on the result of density and FT-IR measurements, as discussed later.

**Figure 3:**
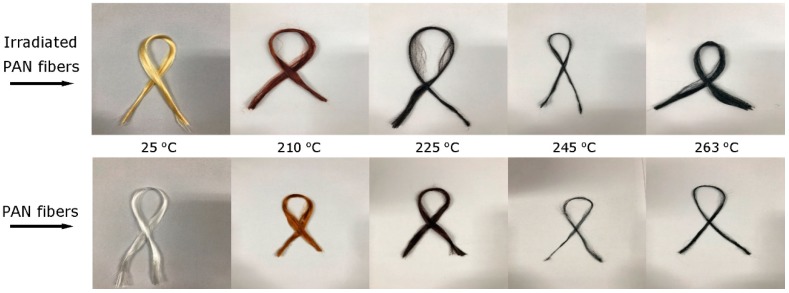
Photographs showing the color change of PAN and irradiated PAN fibers during different stages of stabilization.

Figure 3 shows the color change of PAN fibers after being irradiation and different stages of stabilization. It is clear that the color of PAN fibers changed from white to yellow after e-beam irradiation. Previous studies indicate that the color change of PAN after irradiation was caused by the formation of conjugated double bonds (C=C) or color centers associated with radical species trapped within glassy polymer matrices [10,20]. During the course of stabilization, the PAN precursor fibers underwent a change in color from white to yellow, browns and black; this discoloration at elevated temperature is characteristic of acrylic fibers and is attributed to the conjugation bonds and aromatic structures generated by the oxygen-induced reaction of dehydrogenation [24].

**Figure 4:**
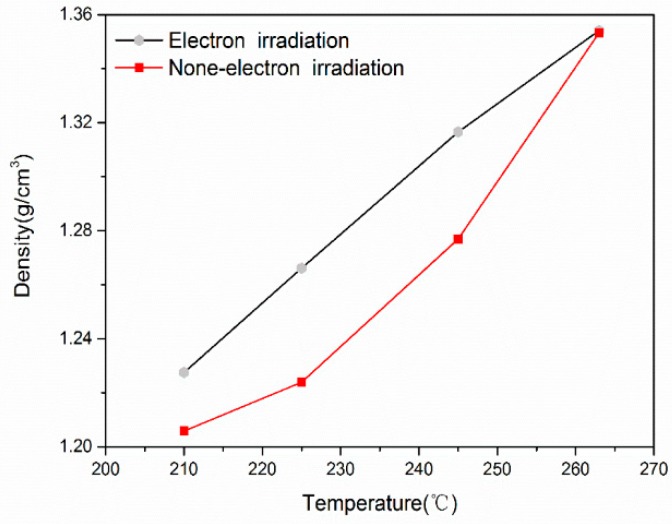
Densities of PAN and irradiated PAN fiber after each stage of stabilization.

The bulk density of fiber is a general reflection of cyclization, dehydration and oxidation reactions during the stabilization process and is commonly used to trace the degree of stabilization reaction [25,26]. The bulk density of PAN fibers before and after irradiation was 1.1871 g/cm^3^ and 1.1925 g/cm^3^, respectively. The slight increase of densities for PAN fibers after irradiation was in agreement with the previous assumptions of irradiation-induced cyclization. For a successful carbonization process, the stabilized fibers should achieve a precursor-dependent critical density [27]. Figure 4 shows the densities of PAN and irradiated PAN fiber after each stage of stabilization. As the stabilization proceeded, the density of stabilized PAN fiber increased. It can be observed that the bulk densities of PAN and irradiated PAN fibers increased gradually with the rise of stabilization temperature. At the early stage of stabilization, the density of irradiated PAN fibers grew faster than PAN fibers, while the density of PAN fibers grew faster at late stages of stabilization. This can be explained by the PAN fibers being pre-stabilized by e-beam irradiation and the densities of irradiated PAN fibers being higher than those of untreated PAN fibers. At later stages of stabilization, the reaction was governed by the thermochemical reaction of remaining PAN molecules. The overall effect was the similar densities of stabilized PAN fibers and stabilized irradiated PAN fibers, which suggested that irradiated PAN fibers with a 24-min stabilization time can achieve the same degree of stabilization compared to PAN fibers with a 40-min stabilization time.

**Figure 5:**
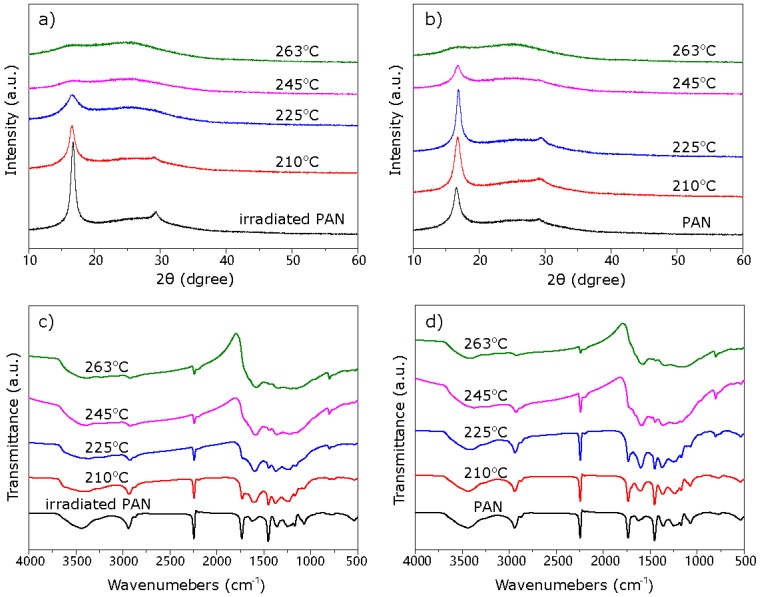
XRD patterns of (**a**) irradiated PAN fibers and (**b**) PAN fibers; FI-IR spectra of (**c**) irradiated PAN fibers and (**d**) PAN fibers at various stages of stabilization.

The crystalline structures of stabilized PAN fibers were verified by XRD patterns, as shown in Figure 5a,b. The diffraction peaks centered at 2θ = 16.8° and 29.2° corresponded to the (100) and (110) crystallographic planes of the PAN quasi-crystals, respectively [23]. The intensity of diffraction peaks at 2θ = 16.8° and 29.2° gradually decreased with the increase of temperature. Simultaneously, there was the appearance of a new diffraction peak at 2θ = 25.5°, which corresponded to amorphous carbon. Meanwhile, for e-beam-irradiated PAN fibers, the diffraction peaks at 2θ = 16.8° and 29.2° disappeared earlier, which indicated that the e-beam irradiation pretreatment can expedite the conversion of the physical stacking structure of PAN molecules.

During oxidative stabilization, two levels/types of structural conversions occurred in precursor fibers: the first one was the conversion of nitrile groups (C≡N) into (C=N) groups, leading to the formation of cyclic structures; while the second one resulted from dehydrogenation and/or oxidation, leading to the formations of aromatic and supra-molecular structures. FT-IR analysis was used to study the effect of irradiation on the chemical structure evolution of PAN fibers, and the results are shown in Figure 5c,d. FI-IR spectra of stabilized PAN fibers contained several important characteristic peaks at 2936 cm^−1^, 2244 cm^−1^ and 1628 cm^−1^, which represented the stretch vibration of methylene (CH_2_), nitrile (C≡N) and (C=N) groups, respectively. With the increase of stabilization temperature, the intensities of C–H and C≡N peaks decreased and the intensities of C=N peak increased, indicating the progress of cyclization reaction. To quantitatively analyze the degree of cyclization reaction during oxidative stabilization, the ring closure index (*RCI*) was measured and is listed in Table 2. It is clear to see that the *RCI*s of PAN fibers increased from 7.85%–18.7% after irradiation at 25 °C, which confirmed that irradiation can affect the chemical structure of PAN through inducing the cyclization reaction. Meanwhile, the *RCI*s of fully-stabilized PAN and irradiated PAN fibers were similar, which supported that a 24-min stabilization of irradiated PAN fibers can achieve the same degree of stabilization compared to 40-min stabilized PAN fibers.

Finally, carbon fibers were prepared based on stabilized PAN and stabilized irradiated PAN fibers, and their mechanical properties are listed in Table 3. The PAN-based carbon fibers (CFs) had a tensile strength of 3.56 GPa and a modulus of 228 GPa, which are standard values for carbon fibers prepared from Courtaulds SAF precursor fibers. The mechanical properties verified that the temperature profiles for stabilization were valid for producing standard carbon fibers. On the other hand, the carbon fibers prepared using irradiated PAN fibers (denoted as i-CFs) had a tensile strength of 2.85 GPa and a modulus of 203 GPa. Although these mechanical properties were lower than carbon fibers prepared from the standard stabilization method, the performance of i-CFs already met the requirements for automobile use. Considering the 40% decrease of stabilization time, the energy consumption and the cost of i-CFs are expected to be noticeably lower than that of standard carbon fibers, making i-CFs competitive for some applications where the balance of the quality and the cost is considered. More importantly, the methods and the materials were all based on a continuous pilot production line, which can provide practical guidance for applying e-beam irradiation to industrial production facilities.

## 4. Discussion

The energy savings of the rapid stabilization process using e-beam irradiation was roughly estimated based on the operation power of the pilot production line in this study. The detailed operation power of the apparatuses is shown in the Supporting Information, Table S1. The stabilization step accounts for ~50% of the total energy consumption of the whole production line. The rapid stabilization process based on e-beam irradiation can save 40% of the energy cost for the thermal stabilization step and save 18% of the total energy cost for the production line. The carbonization process is a relatively fast step that requires less than 3 min. The spinning of PAN fiber is also very fast, and the speed can reach 300–400 m/min. However, the speed of the overall production line has to be limited to around 10–15 m/min due to the long stabilization time. If we can lower the stabilization time, the total production speed can be promoted, and the capacity of each line can be increased; this will enhance the production efficiency and thus further lower the total cost. Meanwhile, the e-beam irradiation of PAN fiber is a fast process, which correlates well with the rapid production of carbon fibers.

It is worth noting that for better comparison of carbon fibers prepared with/without irradiation pretreatment, the current temperature profiles of stabilization were designed for producing standard carbon fibers, which may not be suitable for stabilizing irradiated PAN fibers since there are noticeable differences in the exothermic behavior between irradiated PAN fibers and commercial PAN fibers. Meanwhile, the irradiation dose can be adjusted to further promote the irradiation effect. Thus, there is still room for further decreasing the stabilization time and improving the mechanical properties of i-CFs. Work along these directions is in progress.

## 5. Conclusions

In summary, automobile-grade carbon fibers (12k tow size) with a 2.85 GPa tensile strength and a 203 GPa modulus were continuously prepared with a 24-min stabilization time; and this rapid stabilization of PAN fibers was achieved with the assistance of electron beam irradiation pretreatment. It was shown that e-beam irradiation can lower the onset temperature of the cyclization reaction and mitigate the heat release, which provides the basis for reducing the stabilization time. During the subsequent stabilization process, e-beam irradiation can affect the evolution of both the chemical structure and the crystalline structure of PAN fibers. Finally, judging by the density and the ring closure index, e-beam-irradiated PAN fibers can achieve a comparable degree of stabilization with 40% less time compared to untreated PAN fibers.

## Figures and Tables

**Table 1:** Characterization of DSC curves of PAN and irradiated PAN fibers at various heating rates. *T_onset_*: onset temperature of exothermic reaction; *T_p._*_1_: first exothermic peak temperature; Δ*H*: enthalpy change.

Heating Rate (°C/min)	PAN Fibers			Irradiated PAN Fibers		
	*T_onse_*_t_ (°C)	*T_p._*_1_ (°C)	Δ*H* (J/g)	*T_onset_* (°C)	*T_p._*_1_ (°C)	Δ*H* (J/g)
2	193.7	252.9	3199	186.8	224.2	1666
5	204.8	267.8	2363	189.9	235.1	1258
8	208.3	276.6	1972	181.3	242.6	1584
10	212.6	279.3	1616	182.6	244.9	1309

**Table 2:** The ring closure indexes (*RCIs*) of PAN fibers and irradiated PAN fibers at each stage of thermal stabilization.

	*RCIs*				
	25 °C	210 °C	225 °C	245 °C	263 °C
Irradiated PAN fibers	18.7%	38.3%	61.1%	71.3%	77.5%
PAN fibers	7.85%	22.2%	43.5%	62.7%	76.9%

**Table 3:** Densities and mechanical properties of PAN-based carbon fibers and irradiated-PAN-based carbon fibers with the corresponding coefficient of variance (CV). i-CF, irradiated carbon fiber.

	Density (g/cm^3^)	Tensile Strength (GPa)	CV (%)	Modulus (GPa)	CV (%)	Elongation (%)	CV (%)
CFs	1.805	3.56	2.05	228	2.32	1.51	5.70
i-CFs	1.813	2.85	1.69	203	1.47	1.40	3.47

## Supplementary Materials

The following are available online at http://www.mdpi.com/1996-1944/11/8/1270/s1, Figure S1. Photographs of the experimental setup, Figure S2. Comparison of the DSC curves of PAN and irradiated PAN fibers under different heating rates, Figure S3. DSC curves of PAN and irradiated PAN fibers with baseline, *T_onset_* and *T_p_*_,1_, Table S1. The operation power of the pilot plant.

## References

[1] Frank E., Steudle L.M., Ingildeev D., Spörl J.M., Buchmeiser M.R. (2014). Carbon fibers: Precursor systems, processing, structure, and properties. Angew. Chem. Int. Ed..

[2] Taylor P., Liu Y., Kumar S. (2012). Recent progress in fabrication, structure, and properties of carbon fibers. Polym. Rev..

[3] Salim N.V., Blight S., Creighton C., Nunna S., Atkiss S., Razal J.M. (2018). The role of tension and temperature for efficient carbonization of polyacrylonitrile fibers: Toward low cost carbon fibers. Ind. Eng. Chem. Res..

[4] Huang X. (2009). Fabrication and properties of carbon fibers. Materials.

[5] Sullivan R.A. (2006). Automotive carbon fiber: Opportunities and challenges. JOM.

[6] Baker D.A., Rials T.G. (2013). Recent advances in low-cost carbon fiber manufacture from lignin. J. Appl. Polym. Sci..

[7] Rahaman M.S.A., Ismail A.F., Mustafa A. (2007). A review of heat treatment on polyacrylonitrile fiber. Polym. Degrad. Stab..

[8] Tsai J.-S., Lin C.-H. (1991). Effect of comonomer composition on the properties of polyacrylonitrile precursor and resulting carbon fiber. J. Appl. Polym. Sci..

[9] Dietrich J., Hirt P., Herlinger H. (1996). Electron-beam-induced cyclization to obtain C-fiber precursors from polyacrylonitrile homopolymers. Eur. Polym. J..

[10] Kim S.Y., Lee S., Park S., Jo S.M., Lee H.S., Joh H.I. (2015). Continuous and rapid stabilization of polyacrylonitrile fiber bundles assisted by atmospheric pressure plasma for fabricating large-tow carbon fibers. Carbon.

[11] Liu J., Zhou P., Zhang L., Ma Z., Liang J., Fong H. (2009). Thermo-chemical reactions occurring during the oxidative stabilization of electrospun polyacrylonitrile precursor nanofibers and the resulting structural conversions. Carbon.

[12] Xue Y., Liu J., Liang J. (2013). Correlative study of critical reactions in polyacrylonitrile based carbon fiber precursors during thermal-oxidative stabilization. Polym. Degrad. Stab..

[13] Shin H.K., Park M., Kim H.-Y., Park S.-J. (2015). An overview of new oxidation methods for polyacrylonitrile-based carbon fibers. Carbon Lett..

[14] Ozur G.E., Proskurovsky D.I., Rotshtein V.P., Markov A.B. (2003). Production and application of low-energy high-current electron beams. Laser Part. Beams.

[15] Yuan H., Wang Y., Liu P., Yu H., Ge B., Mei Y. (2011). Effect of electron beam irradiation on polyacrylonitrile precursor fibers and stabilization process. J. Appl. Polym. Sci..

[16] Shin H.K., Park M., Kang P.H., Choi H.-S., Park S.-J. (2014). Preparation and characterization of polyacrylonitrile-based carbon fibers produced by electron beam irradiation pretreatment. J. Ind. Eng. Chem..

[17] Yu H., Yuan H., Wang Y., Wei Z. (2011). The characterization of different doses of electron beam irradiation on the structure and properties of PAN precursor fibers. Adv. Mater. Res..

[18] Shin H.K., Park M., Kim H.-Y., Park S.-J. (2015). Influence of orientation on ordered microstructure of PAN-based fibers during electron beam irradiation stabilization. J. Ind. Eng. Chem..

[19] Kim D.-Y., Shin H.-K., Jeun J.-P., Kim H.-B., Oh S.-H., Kang P.-H. (2012). Characterization of polyacrylonitrile based carbon nanofiber mats via electron beam processing. J. Nanosci. Nanotechnol..

[20] Park S., Yoo S.H., Kang H.R., Jo S.M., Joh H.I., Lee S. (2016). Comprehensive stabilization mechanism of electron-beam irradiated polyacrylonitrile fibers to shorten the conventional thermal treatment. Sci. Rep..

[21] Fitzer E., Frohs W., Heine M. (1986). Optimization of stabilization and carbonization treatment of PAN fibres and structural characterization of the resulting carbon fibres. Carbon.

[22] Shin H.K., Jeun J.P., Kang P.H. (2012). The characterization of polyacrylonitrile fibers stabilized by electron beam irradiation. Fibers Polym..

[23] Lian F., Liu J., Ma Z., Liang J. (2012). Stretching-induced deformation of polyacrylonitrile chains both in quasicrystals and in amorphous regions during the in situ thermal modification of fibers prior to oxidative stabilization. Carbon.

[24] Houtz R.C. (1950). “Orlon” acrylic fiber: Chemistry and properties. Text. Res. J..

[25] Nunna S., Naebe M., Hameed N., Creighton C., Naghashian S., Jennings M.J., Atkiss S., Setty M., Fox B.L. (2016). Investigation of progress of reactions and evolution of radial heterogeneity in the initial stage of thermal stabilization of PAN precursor fibres. Polym. Degrad. Stab..

[26] Liu J., Xiao S., Shen Z., Xu L., Zhang L., Peng J. (2018). Study on the oxidative stabilization of polyacrylonitrile fibers by microwave heating. Polym. Degrad. Stab..

[27] Pethkar S., Dharmadhikari J.A., Athawale A.A., Aiyer R.C., Vijayamohanan K. (2001). Evidence for second-order optical nonlinearity in γ-ray induced partially cross-linked polyacrylonitrile. J. Phys. Chem. B.

