# Supplementary material for: Rapid and Continuous Preparation of Polyacrylonitrile-Based Carbon Fibers with Electron-Beam Irradiation Pretreatment
Source: Materials (Basel). 2018 Jul 24;11(8):1270. doi: 10.3390/ma11081270 (PMC6117685; doi:10.3390/ma11081270)
Supplement: Supplementary file 1 [file materials-11-01270-s001.docx]

Supplement

Rapid and Continuous Preparation of Polyacrylonitrile-Based Carbon Fibers with
Electron-Beam Irradiation Pretreatment

Jia Yang ^1^, Yuchen Liu ^1^, Jie Liu ^1,^*, Zhigang Shen ^2^, Jieying Liang ^1^ and Xiaoxu Wang ^1,^*

^1^ Key Laboratory of Carbon Fiber and Functional Polymers, Ministry of Education, Beijing University of Chemical Technology, Chao-Yang District, Beijing 100029, China; yangjcarbon@163.com (J.Y.); 13701098376@163.com (Y.L.); liangjy@mail.buct.edu.cn (J.L.)

^2^ SINOPEC Shanghai Research Institute of Petrochemical Technology, 1658 Pudong North Road,
Pudong District, Shanghai 201208, China; shenzg.sshy@sinopec.com

***** Correspondence: liuj@mail.buct.edu.cn (J.L.); wangxiaoxu@mail.buct.edu.cn (X.W.);
Tel.: +86-10-6443-8724 (J.L. & X.W.)

Received: 19 June 2018; Accepted: 18 July 2018; Published: date


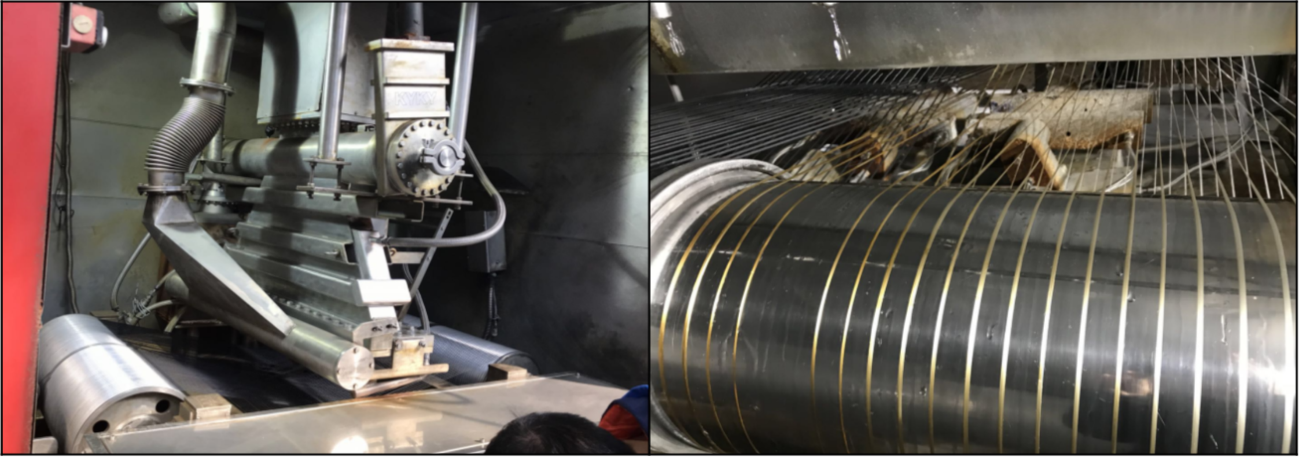


**Figure S1.** Photographs of the irradiation setups.


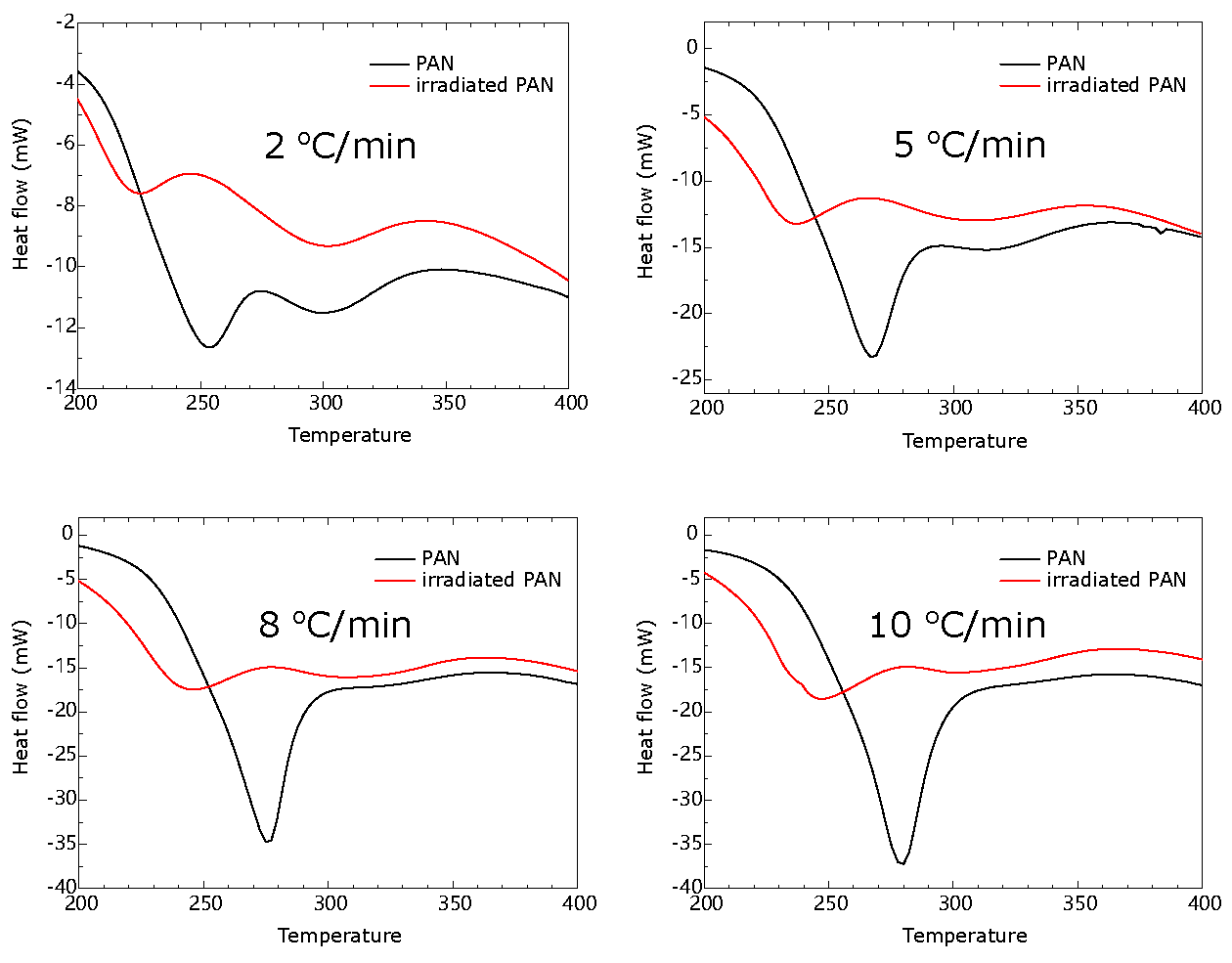


**Figure S2.** Comparison of DSC curves of PAN and irradiated PAN fibers under different heating rate.


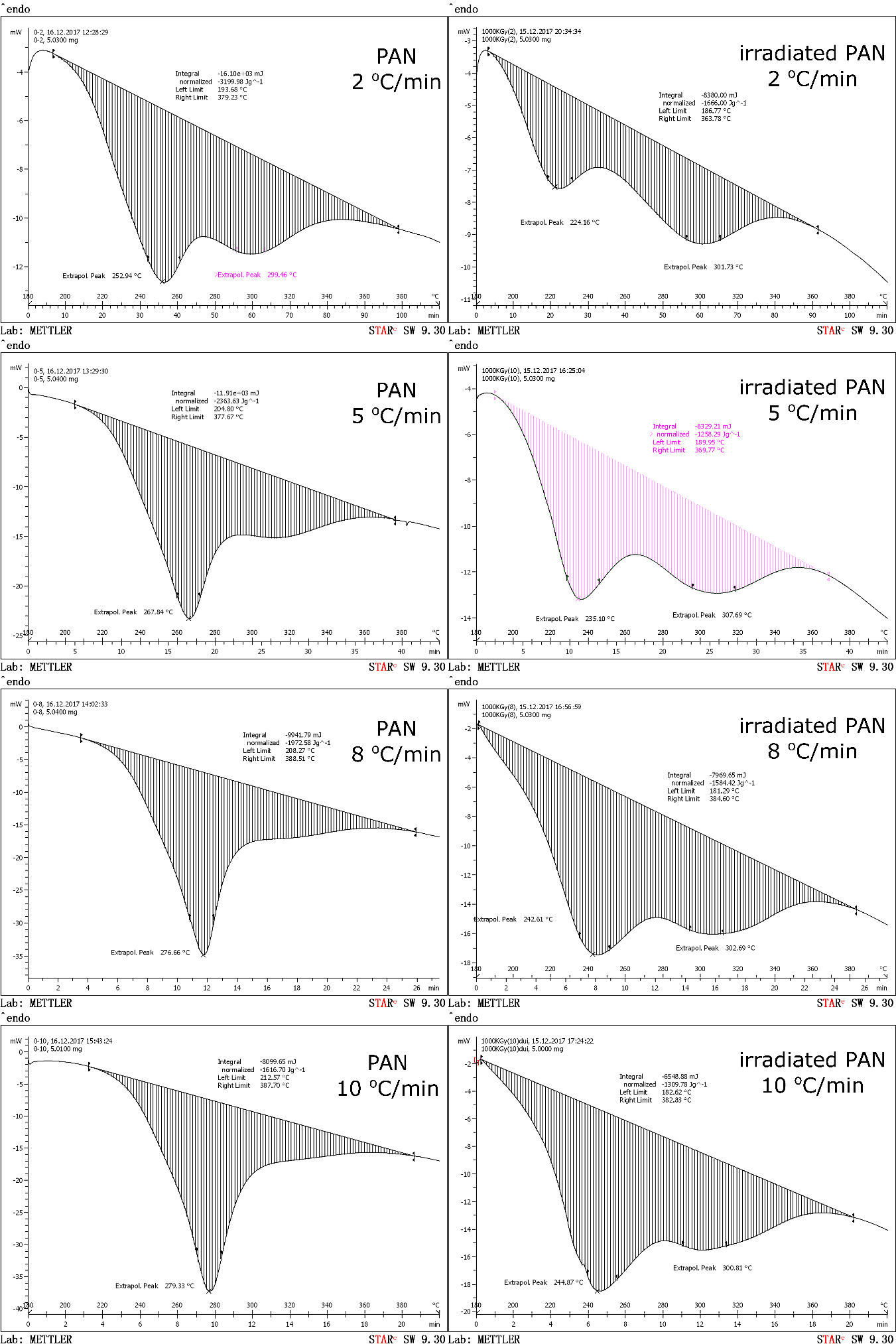


**Figure S3.** DSC curves of PAN and irradiated PAN fibers with baseline, *T_onset_* and *T_p,_*_1_.

**Table S1.** The operation power of the pilot plant.

|  | E-beam accelerator (KW) | Stabilization furnaces (KW) | Low-temperature carbonization furnace (KW) | High-temperature carbonization furnace (KW) | Drivers (KW) | Total (KW) |
| --- | --- | --- | --- | --- | --- | --- |
| 40-min stabilization |  | 67 | 30 | 20 | 10 | 127 |
| 24-min stabilization | 3.5 | 40 | 30 | 20 | 10 | 103.5 |

© 2018 by the authors. Submitted for possible open access publication under the terms and conditions of the Creative Commons Attribution (CC BY) license (http://creativecommons.org/licenses/by/4.0/).
